# Psychosocial Predictors for Cancer Prevention Behaviors in Workplace Using Protection Motivation Theory

**DOI:** 10.1155/2015/467498

**Published:** 2015-10-12

**Authors:** Mohammad Javad Zare Sakhvidi, Maryam Zare, Mehrdad Mostaghaci, Amir Houshang Mehrparvar, Mohammad Ali Morowatisharifabad, Elham Naghshineh

**Affiliations:** ^1^Department of Occupational Health, Faculty of Health, Shahid Sadoughi University of Medical Sciences, Yazd, Iran; ^2^Department of Occupational Health, Faculty of Health, Shahid Sadoughi University of Medical Sciences, International Campus, Yazd, Iran; ^3^Department of Occupational Medicine, Faculty of Medicine, Shahid Sadoughi University of Medical Sciences, Yazd, Iran; ^4^Department of Health Education, Faculty of Health, Shahid Sadoughi University of Medical Sciences, Yazd, Iran; ^5^Department of Obstetrics and Gynecology, Isfahan University of Medical Sciences, Isfahan, Iran

## Abstract

*Backgrounds.* The aim of this study was to describe the preventive behaviors of industrial workers and factors influencing occupational cancer prevention behaviors using protection motivation theory. *Methods.* A self-administered questionnaire was completed by 161 petrochemical workers in Iran in 2014 which consisted of three sections: background information, protection motivation theory measures, and occupational cancers preventive behaviors. *Results.* A statistically significant positive correlation was found between PM and self-efficacy, response efficacy, and the cancer preventive behaviors. Meanwhile, statistically significant negative correlations were found between PM, cost, and reward. *Conclusions.* Among available PMT constructs, only self-efficacy and cost were significant predictors of preventive behaviors. Protection motivation model based health promotion interventions with focus on self-efficacy and cost would be desirable in the case of occupational cancers prevention.

## 1. Introduction

Cancer is the leading cause of death, with annual 8.2 million deaths globally, which imposes a heavy health and economic burden on the society, especially in developing and underdeveloped countries [1]. International Agency for Research on Cancer (IARC) has classified about 114 agents, mixtures, and exposure situations as known or probable human carcinogens [2]. Many proven human carcinogens (IARC group 1) are from occupational origin. About 19% of cancers are attributed to environmental factors [3]. Despite clinical and biological analogy between occupational and nonoccupational cancers, long latency period and lack of knowledge about their actual etiology lead to late diagnosis and high mortality among the patients [4]. Nearly all of the occupational cancers are preventable by identifying and controlling the exposures. A fact about occupational cancers is that all of them have controllable causes and hence are potentially preventable [5]. According to World Health Organization (WHO), the primary preventions are the vital steps in the global control of occupational and environmental cancers [6]. Health education is a cost-effective and desirable approach to prevent the exposure to etiologic factors and hence to reduce the global burden of occupational cancers.

Promotion of protective behaviors in the workplace as a cost-effective method is the most important element in cancer prevention. There are different types of protective behaviors available to workers to employ during their routine jobs. These behaviors include usage of personal protective equipment (PPE), minimizing exposure to carcinogens, engineering controls, and regular medical surveillance. However, most studies reported a low level of protective behaviors in workers and suggest that focus should be on the implications to enhance protective behaviors [7, 8].

Preventive behavior toward cancer prevention in workers is a multifactorial phenomenon and depends on large numbers of demographic, socioeconomic, and psychological factors [8]. There are numerous theories used to predict intention for health behaviors. Protection motivation theory (PMT) is extensively used in the study of cancer prevention behaviors such as skin cancer risk reduction through an intervention in a group of college students, study of cancer prevention as a source of exercise motivation [9, 10], and intention to use genetic testing for breast cancer risk [11]. Based on PMT, a man's behavior toward a defined health hazard depends on* threat appraisal* and* coping appraisal* [12]. Threat appraisal is a measure of severity of hazard and vulnerability to it, and coping appraisal is a measure of response to hazardous situation. The threat appraisal comprises 3 scales including* perceived reward* (positive aspects of doing unhealthy behaviors),* perceived vulnerability* (probability of personally experiencing the harm from a defined unhealthy behavior), and* perceived severity* (perceived degree of harm from an unhealthy behavior) of hazard. The coping appraisal comprises* self-efficacy* (belief about ability to perform protective measures),* response efficacy* (effectiveness of the proposed protective measure in risk reduction), and* response costs* (expected costs associated with a healthy behavior). The combination of threat appraisal and coping appraisal forms the protection motivation (PM) for healthy behavior [12].

In this study, we evaluated the applicability of PMT to predict cancer protective behaviors of a group of petrochemical workers against exposure to occupational carcinogens in the workplace in Iran. The results of this study can be used in interventional programs aimed at promoting occupational cancer preventive behaviors in the workers. The aim of this study was to describe the preventive behaviors of industrial workers and factors influencing occupational cancer prevention behaviors using PMT framework. Study objectives included the following: to examine the pattern of preventive behaviors against occupational cancers in Iranian industrial workers; to examine the role of demographic characteristics, on PM and occupational cancer preventive behaviors; to identify the predictive role of various PMT measures on PM in industrial workers; and to identify factors associated with Iranian industrial workers' occupational cancers preventive behaviors.

## 2. Methods

### 2.1. Study Design and Subjects Selection

This is a cross-sectional study on a sample of petrochemical workers performed in Asalouyeh special petrochemical zone, South Pars gas field, Bushehr, Iran, in 2014. Participants were selected from workers of a large urea and ammonia production facility. According to risk assessments conducted by industrial hygienists, there was potential exposure to numerous carcinogenic compounds and agents such as benzene, polyaromatic hydrocarbons, inorganic acids, cutting oils, and ultraviolet radiation. Sample size was calculated based on the literature about prediction of sun protective behaviors using PMT variables [13]. Correlation coefficients between PM and measures of PMT were used in sample size equation with desired significance level of 1% and power of 85% [14]. Based on the desired assumptions, we need a sample size of 175 subjects for two tiled tests. All samples were selected from subjects who had direct contact with process or direct engagement in construction phases. Those who worked in the administrative offices were excluded from the study. Participants were informed about the study objectives and entered into the study voluntarily.

### 2.2. Measures

#### 2.2.1. Demographic Section

Demographic characteristics including age, sex, work history, daily work hours, marital status, family size, educational level, and income levels were asked in the first section of the questionnaire. History of malignant diseases and its consequent death in the family or friends of participants was asked separately.

#### 2.2.2. PMT Measures

In the second section, PMT constructs including rewards, severity, vulnerability, response efficacy, self-efficacy, and response costs were measured. Content and face validity of PMT questionnaire were confirmed by a group of experts (two academic staff in health promotion and three occupational hygienists). The items were scored on a 5-point Likert scale from 1 (completely disagree) to 5 (completely agree). Fifteen workers participated in a pilot study to determine the internal consistency of constructs. Cronbach's alpha of the constructs ranging from 0.676 to 0.846 showed an acceptable internal consistency.  Table 1 shows the detailed description of PMT constructs.

*(i) Vulnerability.* Vulnerability measures how a susceptible participant is at risk of developing health threat (e.g., cancer would result from my conditions).

*(ii) Severity.* We assessed participants' perceived severity of occupational cancer risk using four items. It measured how seriously the worker took the health threats. Higher perceived severity could be a good predictor for motivation to adopt a protective behavior.

*(iii) Self-Efficacy.* Self-efficacy was defined as the belief or level of confidence in one's ability to successfully perform the recommended preventive behavior (e.g., I can protect myself from excessive exposure to carcinogens in the workplace).

*(iv) Response Efficacy.* Response efficacy was measured by 5 items. Response efficacy measures one's ability to perform preventive or protective behaviors which will be effective in risk reduction (e.g., I can reduce the risk of cancer in the workplace by proper use of personal protective equipment).

*(v) Fear of Cancer.* Six items of “cancer fear scale” also were used to assess the cancer fear in participants.

*(vi) Perceived Rewards.* Perceived rewards are about positive feeling toward doing unhealthy behaviors (e.g., when I did not use safety and health precautions in working with chemicals, it looks like I am more experienced).

*(vii) Perceived Cost.* Perceived costs associated with recommended protective behavior were regarded as response costs (e.g., it is time consuming for me to use personal protective equipment). It was measured by 6 items.

#### 2.2.3. Behaviors

Occupational cancer preventive behaviors were assessed by seven items developed and validated by the authors. Questions were composed of different types of available preventive options including use of personal protective equipment (PPE), application of engineering controls (use of local ventilation), seeking chemical safety data prior to use (via checking chemical labels and material safety data sheets (MSDSs)), and regular medical checkups. On a 5-point Likert scale (1 = always; 5 = never), participants reported the extent to which they perform protective measures in the statements. The items demonstrated high internal consistency (*α* = 0.814).

### 2.3. Data Preparation and Statistical Analysis

SPSS software package version 20 (SPSS, Inc., Chicago, IL) was used for statistical analysis. Descriptive statistics were performed over all demographic, background information and constructs. Quantitative variables were described by mean and standard deviation. Categorical variables were presented as number and percent (*N*, %). Univariate comparisons were performed by *t*-test. Pearson's correlation test was used to evaluate the correlation between PMT constructs. To assess the predictability of PMT constructs over and above the influence of other parameters, hierarchical multiple linear regression was performed [15]. *Z* scores of variables were calculated and were entered into the regression analysis. Level of significance was set at *p* value < 0.05. Regression results were checked for level of collinearity.

## 3. Results

From 180 workers approached, 161 workers agreed to participate (89.4%) and completed the questionnaire. Those who worked in administrative offices were excluded from the study. Participants ranged in age from 22 to 55 years (*M* = 32.21, SD = 4.22) with mean family size of 3.16 (SD = 1.61) and 11.69 hours of daily work (SD = 1.27). Mean duration of employment of participants was 6.64 years (SD = 2.90). Most participants were males (60.9%), had completed at least some college or specialized training (88.8%), were married (83.1%), and had a gross monthly income greater than 600 USD (92.7%). See Table 2 for detailed demographic information.

### 3.1. Cancer Protective Behaviors

**Figure 1:**
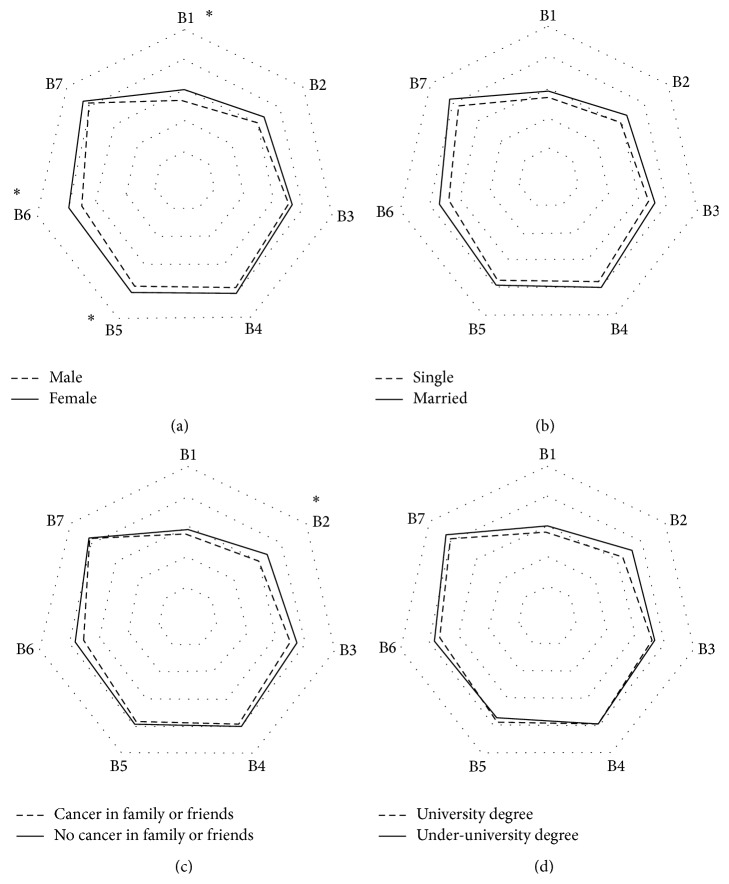
Radar chart depicting mean total score of each preventive behavior according to (a) gender, (b) marital status, (c) history of cancer in family or friends, and (d) educational status. B1–B7: different behaviors according to coding in Table 3. *∗* means significant difference in mean values of behavior in two groups (*p* < 0.05).

Table 3 shows the frequency of cancer protective behaviors reported by the study participants.  Table 5 also shows the behavior scores according to different demographic characteristics. Among the preventive measures, asking the information regarding hazardous chemicals from occupational safety and health (OSH) person and study about OSH issues of carcinogens were reported to be the lowest, and periodic medical checkup was reported to be the most. Total behavior score (sum of all behavior attributes) in female workers (*M* = 26.34; SD = 4.61) was statistically higher than male workers (*M* = 24.29; SD = 4.69) (*p* = 0.015). Further analysis on each of the behaviors showed that females had higher scores in asking information from OSH person (B1), use of engineering control options (B5), and use of PPE (B6). Married workers (*M* = 25.55; SD = 4.61) also showed higher but not statistically significant behavior score in comparison with singles (*M* = 23.89; SD = 4.94). There was also no marital status specific difference in each of the single behavior attributes. There was no statistical difference either in behavior score or in each single behavior attribute in terms of education (*p* = 0.418 for total behavior score). Those who reported history of cancer in those relatives or friends also showed no difference in total preventive score; however, when the analysis was performed for each of the behavior attributes separately, study about OSH issues of carcinogens in the group with history of cancer in their relatives or friends was significantly higher than other group.  Figure 1 shows the radar chart of each of the behavior scores according to different demographical characteristics of participants.

### 3.2. Correlational Results of PMT Constructs

Pearson's product-moment coefficients among the PMT constructs and age are shown in Table 4. For gender, the results are based on Spearman's rank correlation. A statistically significant positive correlation was found between PM and self-efficacy, response efficacy, and the cancer preventive behaviors. Meanwhile, statistically significant negative correlations were found between PM, cost, and reward. A pattern similar to PM was also observed for behavior. Behavior was also significantly correlated with gender. Response efficacy was positively correlated with self-efficacy and negatively with severity, fear, cost, and reward. Fear was positively correlated with vulnerability and severity. Self-efficacy was negatively associated with severity, vulnerability, and fear. Results showed that PM is not significantly different in terms of gender, marital status, educational level, cancer history in family, and cancer history in friends (Table 5). The results indicated very low level of collinearity according to VIF statistics.

### 3.3. Efficiency of PMT Measures

Hierarchical multiple linear regressions were performed in two blocks to assess the efficiency of PMT constructs over the influence of other parameters. Predictors were classified in two different blocks according to their natures:

1. Demographic characteristics block: sex, age, marital status, education, and work history.
2. PMT block: this block comprised 7 different constructs of PMT.

Demographic characteristics of participants explained 1.9% of the observed variance in PMT which was not significant at 0.05 level (Table 6). However, PMT constructs were responsible for 22.1% change in observed variance which was statistically significant (*p* < 0.001). Other hierarchical multiple linear regressions were also performed with “cancer preventive behaviors” as a dependent variable (Table 7). In the first block, only gender had significant effect on preventive behaviors; however, under influence of PM constructs, none of the demographic variables had significant effect on preventive behaviors. In the second block, self-efficacy and perceived costs were significant predictors of preventive behaviors. However, self-efficacy was the most strong and positive predictor, and perceived cost was significant negative predictor of preventive behavior.

## 4. Discussion

The study investigated the applicability of PMT variables as predictors of occupational cancers behavioral intentions and preventive behaviors in a group of Iranian industrial workers. The total cancer preventive score in participants was above the average. This finding could be due to the high quality of OSH services and trainings in the petrochemical industries in Iran. Among preventive behaviors, periodical medical checkups had the highest score which was possibly due to the availability of the services freely in the industrial complex. Availability of health services leads to less perceived barrier to conduct adaptive behaviors and therefore is an influential parameter in promotion of safe behavior of people toward health hazards [16]. Technical protective behaviors such as limiting spillage or use of engineering controls received second and third ranks in protective behaviors. However, behaviors which need interpersonal communication (asking chemical hazard information from OSH person) gained the lowest attention. Costumer's perceived reliability and empathy from OSH person or office have a role in consumers' perception and then after conduct organizational advices. No difference was observed across educational levels regarding the total score of preventive actions. This implies that, in the situations with a high quality of health services, all employees are interested in protective behaviors regardless of their education. Availability of health services and their quality diminish the role of such demographical factors as education. However, our findings should be interpreted cautiously, because about 89% of our participants have graduated from a university and only one case had not finished high school education. We found no significant difference between cancer preventive behaviors with regard to marital status. Family support is among the most important predictors of preventive behaviors, but we did not measure family support, and our results could be only used as a surrogate of support. Being married does not necessarily mean high level of family support. It also may depend on the type of disease. In the case of mammography for breast cancer, the family acceptance was the strongest predictor of mammography [17]. Another important issue which should be considered is the type of residency in most of the petrochemical workers in our study. Most of our participants live in camps away from their family for 14 or 21 consecutive days. This lifestyle may also reduce the role and effect of family on healthy behaviors. Preventive behaviors score was gender dependent in the present study. Women had a significantly higher total score of preventive behavior than men. In general, men are more risky than women. Several other studies reported such findings about the difference between genders regarding health beliefs and behaviors in other ethnicities [18, 19]. This finding suggests that gender specific health promotion programs should be planned to promote cancer preventive behaviors in industrial workers.

Our findings about predictors of PM and preventive behaviors are in accordance with PMT framework. In general, response efficacy and self-efficacy were positively correlated with PM and preventive behaviors. Perceived adaptive response cost and maladaptive response reward also negatively affect PM and preventive behaviors [12]. Surprisingly, components of coping appraisals were the strongest predictors of behavioral intention in workers. Self-efficacy was the strongest predictor of PM followed by response efficacy. Workers with high self-efficacy believe that they have the ability to succeed; therefore, they tend to conduct healthy behavior when the encounter hazardous situations. Highly educated workers' and higher knowledge level about the source of occupational cancer in their workplace may be the cause of high rating of their ability and response efficacy [20]. Improving self-efficacy can improve the preventive behaviors in vulnerable population [21, 22]. Health promotion programs aimed at improvement of cancer preventive behaviors self-efficacy may promote the behavior of at-risk workers.

Level of cancer fear in this study was relatively high. Several other researchers found that people with a high risk situation of developing a cancer had a relatively higher score of cancer fear [23]. However, there is no consistency between the results, and recent larger studies found low to moderate cancer fear in at-risk populations. It was positively influenced by perceived severity and vulnerability and negatively by self-efficacy and response efficacy. But it was not a significant predictor of PM or preventive behaviors. Fear has mediating effects on preventive behavior and affects behavior just in the specific situation of self-efficacy and response efficacy. In our study, coping appraisal score is relatively higher than threat appraisal score and therefore it would be the best description for the finding. It is also in accordance with McGinty et al. who found that high threat appraisal in combination with low coping appraisal is related to the fear of cancer in survivors of breast cancer [24].

In our previous study, we found an optimistic bias in response of workers to the questions about their susceptibility to developing occupational cancer. This may be the explanation of our recent finding on the nonsignificant effect of threat appraisals (vulnerability and severity) on the PM and preventive behavior of participants. In the case of severity, it is a relatively abstract notation and participants are not aware of the exact pattern of threat [13]. Perceived severity is probably an age dependent measure, and in older subjects it is higher than in young adults [25]. In the case of preventive behaviors toward breast cancer, perceived severity was a predictor of the intention to perform breast self-examination [26]. However, we found no correlation between severities in either behavior or PM. Effect size of coping or threat variables on protective behaviors to some extent depend on the nature of health problem. According to other studies, threat appraisal variables are the strongest predictors of cancer preventive behaviors; however, for smoking, coping appraisals are the strongest variables in predicting preventive behaviors [12]. In this study, most of our participants were young workers (about 82% of them were less than 35 years old) and this may be the main cause of our findings about nonsignificant effect of perceived severity and vulnerability on the PM and preventive behaviors.

Perceived cost was also a significant predictor of PM and preventive behaviors. The results suggest that, among vulnerable subjects, perceived cost of doing healthy behaviors is more important than awareness of rewards related to maladaptive behaviors. However, in the case of other healthy behaviors such as condom use, perceived rewards were more important than perceived costs [27].

### 4.1. Study Strength

The present research has several strengths. First of all, it is one of the few studies specifically pertained to application of cognitive models in occupational cancer preventive behaviors in industries. There are numerous studies on the application of models on cancer preventive measures, but to the best of our knowledge, none of them were applied in the industrial zone and focused on occupational cancers. Our findings are an addition to the current literature about influential parameters on safe and healthy behaviors toward occupational cancers in industrial work environments. Second, in addition to simple mean difference statistics and correlation matrix, we employed hierarchical multiple linear regression to test efficiency of the PMT constructs in the prediction of PM and behavior. This approach was successfully applied by other researchers before.

## 5. Limitations

This study has some limitations which should be considered in the future studies. First, the data were based on self-reports which may be subject to over- or underreporting, potentially distorting the results. Use of more objective measures such as biological markers of exposure would be an interesting idea and is proposed for future studies. Second, this study was conducted in an industrial sector with a relatively high quality of occupational health and safety services, young workforces, and relatively educated workers. Therefore, the generalization of the results should be made with caution. The level of health and safety culture can be highly variable from company to company, and it also depends on sociocultural differences. It would be worth exploring the applicability of PMT (and determining the predictive variables of cancer prevention) in other cultures and companies. The questionnaires used in this study to assess cancer preventive behaviors and elements of protection motivation were self-developed by research group. However, considering the reliability and validity of the assessments through statistical analysis, pilot study, and panel of experts, the results should be used with caution in different societies.

## 6. Conclusion

In general, PM was a strong predictor of preventive behaviors. Among available PMT constructs, only self-efficacy and cost were significant predictors of preventive behaviors. PMT based health promotion interventions with focus on self-efficacy and cost would be desirable in the case of occupational cancers prevention. Role of family support in industrial camping residency should be considered and needs more research.

## Figures and Tables

**Table 1:** Characteristics of PMT constructs questionnaire.

Construct	Item number	*α*	Possible range
Perceived severity	4	0.770	4–20
Perceived vulnerability	3	0.715	3–15
Perceived rewards	2	0.676	2–10
Self-efficacy	8	0.804	8–40
Response efficacy	5	0.70	5–25
Response costs	6	0.706	6–30
Fear	6	0.846	6–30
Protection motivation	4	0.815	4–20

**Table 2:** Sample characteristics (*N* = 161) (missing are excluded from calculations).

Variable	Mean (SD) or *N* (%)
Age	32.21 (4.22)
Gender	
Male	98 (60.9)
Female	63 (39.1)
Work experience	6.64 (2.90)
Marital status	
Single	27 (16.9)
Married	133 (83.1)
Divorced	1 (0.62)
Family size	3.16 (1.61)
Daily work hours	11.69 (1.27)
Education	
Less than high school	1 (0.6)
Graduated from high school	17 (10.6)
University degree	142 (88.8)
Income	
Less than 300 US$	4 (2.6)
300–600 US$	7 (4.7)
Above 600 US$	143 (92.7)
Cancer in friends or coworkers	
Yes	55 (34.6)
No	104 (65.4)
Cancer in family or relatives	
Yes	64 (40)
No	96 (60)
Cancer death in friends or coworkers	
Yes	50 (31.4)
No	109 (68.6)
Cancer death in family or relatives	
Yes	64 (40)
No	94 (58.8)

**Table 3:** A Summary of protective behaviors frequencies among participants (*n* = 161) *N* (%).

Protective behaviors	Never	Seldom	Sometimes	Mostly	Always	Item mean	Item SD
Asking information from HSE person (B1)	16 (10)	53 (33.12)	45 (28.12)	33 (20.62)	13 (8.12)	2.84	1.12
Study about OSH of carcinogens (B2)	6 (3.75)	29 (18.12)	67 (41.87)	42 (26.25)	16 (10)	3.21	0.98
Considering chemical labels (B3)	4 (2.5)	22 (13.75)	42 (26.25)	64 (40)	28 (17.5)	3.56	1.01
Lowering chemical spillage (B4)	1 (0.625)	8 (5)	32 (20)	72 (45)	47 (29.37)	3.98	0.87
Use of engineering control options (B5)	2 (1.25)	6 (3.75)	46 (28.75)	63 (39.37)	43 (26.87)	3.87	0.90
Use of PPE (B6)	2 (1.25)	16 (10)	50 (31.25)	61 (38.12)	31 (19.37)	3.64	0.95
Periodic clinical checkup (B7)	5 (3.12)	9 (5.62)	24 (15)	44 (27.5)	78 (48.75)	4.13	1.06

**Table 4:** Descriptive statistics and intercorrelations between the main measures (*N* = 162).

	Age	Gender	Vulnerability	Severity	Fear	Self-efficacy	Cost	Reward	Response efficacy	Protection motivation	Behavior
Age	—	.029	−.127	−.066	−.090	−.040	−.125	−.054	−.074	.084	.099
Gender		—	−.002	.077	−.100	.112	−.127	−.140	.065	.093	.193^*∗*^
Vulnerability			—	.524^*∗∗∗*^	.547^*∗∗∗*^	−.226^*∗∗*^	.147	−.146	−.068	.084	.023
Severity				—	.604^*∗∗∗*^	−.356^*∗∗∗*^	.213^*∗∗*^	−.051	−.200^*∗*^	.007	−.007
Fear					—	−.347^*∗∗∗*^	.090	.038	−.181^*∗*^	−.011	−.091
Self-efficacy						—	−.229^*∗∗*^	−.232^*∗∗*^	.570^*∗∗∗*^	.425^*∗∗∗*^	.372^*∗∗∗*^
Cost							—	.062	−.197^*∗*^	−.244^*∗∗*^	−.289^*∗∗∗*^
Reward								—	−.282^*∗∗∗*^	−.270^*∗∗∗*^	−.228^*∗∗*^
Response efficacy									—	.447^*∗∗∗*^	.294^*∗∗∗*^
Protection Motivation										—	.517^*∗∗∗*^
Behavior											—
Mean	32.21	98^a^	12.96	16.06	21.89	26.41	17.44	4.01	18.48	15.35	25.23
SD	4.22	60.9^b^	1.94	2.88	4.77	5.23	3.77	1.37	3.18	2.67	4.72

^*∗*^Correlation is significant at the 0.05 level (2-tailed).

^*∗∗*^Correlation is significant at the 0.01 level (2-tailed).

^*∗∗∗*^Correlation is significant at the 0.001 level (2-tailed).

^a^Number; ^b^(%) of males.

**Table 5:** Comparison of the protection motivation mean score and cancer preventive behaviors based on some demographic variables (*n* = 161).

Demographic variable	Level	Protection motivation		Cancer preventive behaviors	
		Mean (SD)	*p* value	Mean (SD)	*p* value
Gender	Male	15.15 (2.72)	0.245	24.49 (4.61)	0.015
	Female	15.65 (2.59)		26.35 (4.69)	

Marital status	Single	14.96 (3.50)	0.423	23.89 (4.94)	0.094
	Married	15.42 (2.50)		25.55 (4.61)	

Education	School	15.18 (2.35)	0.797	26.06 (4.49)	0.418
	University	15.35 (2.72)		25.08 (4.73)	

Cancer history in friends	Yes	15.04 (3.04)	0.226	24.16 (5.27)	0.035
	No	15.57 (2.37)		25.83 (4.32)	

Cancer history in family	Yes	15.65 (2.84)	0.250	24.88 (4.58)	0.460
	No	15.14 (2.57)		25.44 (4.84)	

**Table 6:** Predicting protection motivation: hierarchical regression analysis (*N* = 161).

Step/variable	*β* (Step 1)	*β* (Step 2)
(1) Age	0.185	0.151
Gender	0.143	.042
Duration of employment	−0.116	−.037
Education	−.042	−.012
Marital status	0.108	.073
(2) Fear		.037
Self-efficacy		0.258^*∗∗*^
Cost		−0.164^*∗*^
Response efficacy		0.218^*∗*^
Reward		−0.121
Vulnerability		0.113
Severity		0.101
Δ*R* ^2^	0.019	0.221
Cumulative Δ*R* ^2^	0.019	0.240
*p*	0.178	0.0001

^*∗*^Significant at 0.05 level.

^*∗∗*^Significant at 0.01 level.

**Table 7:** Predicting cancer preventive behaviors: hierarchical regression analysis (*N* = 161).

Step/variable	*β* (Step 1)	*β* (Step 2)
(1) Age	.159	.103
Gender	.184^*∗*^	.079
Duration of employment	−.111	−.033
Education	−.085	−.060
Marital status	.135	.111
(2) Fear		−.117
Self-efficacy		.266^*∗*^
Cost		−.205^*∗*^
Response efficacy		.054
Reward		−.075
Vulnerability		.080
Severity		.180
Δ*R* ^2^	0.040	0.124
Cumulative Δ*R* ^2^	0.040	0.164^*∗∗∗*^
*p*	0.060	0.001

^*∗*^Significant at 0.05 level.

^*∗∗∗*^Significant at 0.001 level.
